# Proposal of a new tool for paediatric primary spontaneous pneumothorax: the Spontaneous Pneumothorax in Adolescents and Children Evaluation (SPACE) score

**DOI:** 10.3389/fped.2026.1921834

**Published:** 2026-08-27

**Authors:** Simone Frediani, Ivan Pietro Aloi, Angelo Zarfati, Filomena Valentina Paradiso, Valerio Pardi, Sara Silvaroli, Antonella Accinni, Federico Beati, Arianna Bertocchini, Silvia Madafferi, Giovanni Rollo, Alessandro Inserra, Lorenzo Nanni

**Affiliations:** 1 General and Emergency Pediatric Surgery Unit, Bambino Gesù Children’s Hospital, IRCCS, Rome, Italy; 2 Pediatric Surgery, IRCCS, Policlinico Universitario A. Gemelli, Rome, Italy

**Keywords:** children, pediatric, primary spontaneous pneumothorax, score, thoracic drainage

## Abstract

**Background and aims:**

Primary Spontaneous Pneumothorax (PSP) in paediatric patients is often managed according to adult guidelines, which may be inappropriate due to anatomical and physiological differences between adults and children. No management score has ever been developed for paediatric PSP. The aim of the study was to present the Spontaneous Pneumothorax in Adolescents and Children Evaluation (SPACE) score, a clinical scoring system designed to enhance decision-making in the paediatric PSP management.

**Methods:**

This bicentric retrospective study was conducted across two tertiary paediatric surgery centres, including 53 paediatric patients diagnosed with their first PSP episode (Period: January 2012-January 2025). This study evaluated clinical and radiological factors influencing treatment decisions and employed statistical analyses to develop and validate the SPACE Score. The exclusion criteria encompassed inadequate data, loss to follow-up, and age exceeding 18 at the time of diagnosis.

**Results:**

Significant differences in PSP dimensions and clinical outcomes were observed between conservative (*n* = 35) and drainage (*n* = 18) treatment groups. The SPACE score demonstrated excellent predictive accuracy in distinguishing treatment needs, with area under the receiver operating characteristic curve values for all models exceeding 0.90. The presence of mediastinal shift and pneumothorax dimensions were identified as critical predictors of the need for drainage.

**Conclusions:**

The SPACE score is a potential tool for improving paediatric PSP management, providing a standardized approach to treatment decisions. However, further research is needed to improve its applicability across clinical settings.

## GRAPHICAL ABSTRACT

**Figure:**
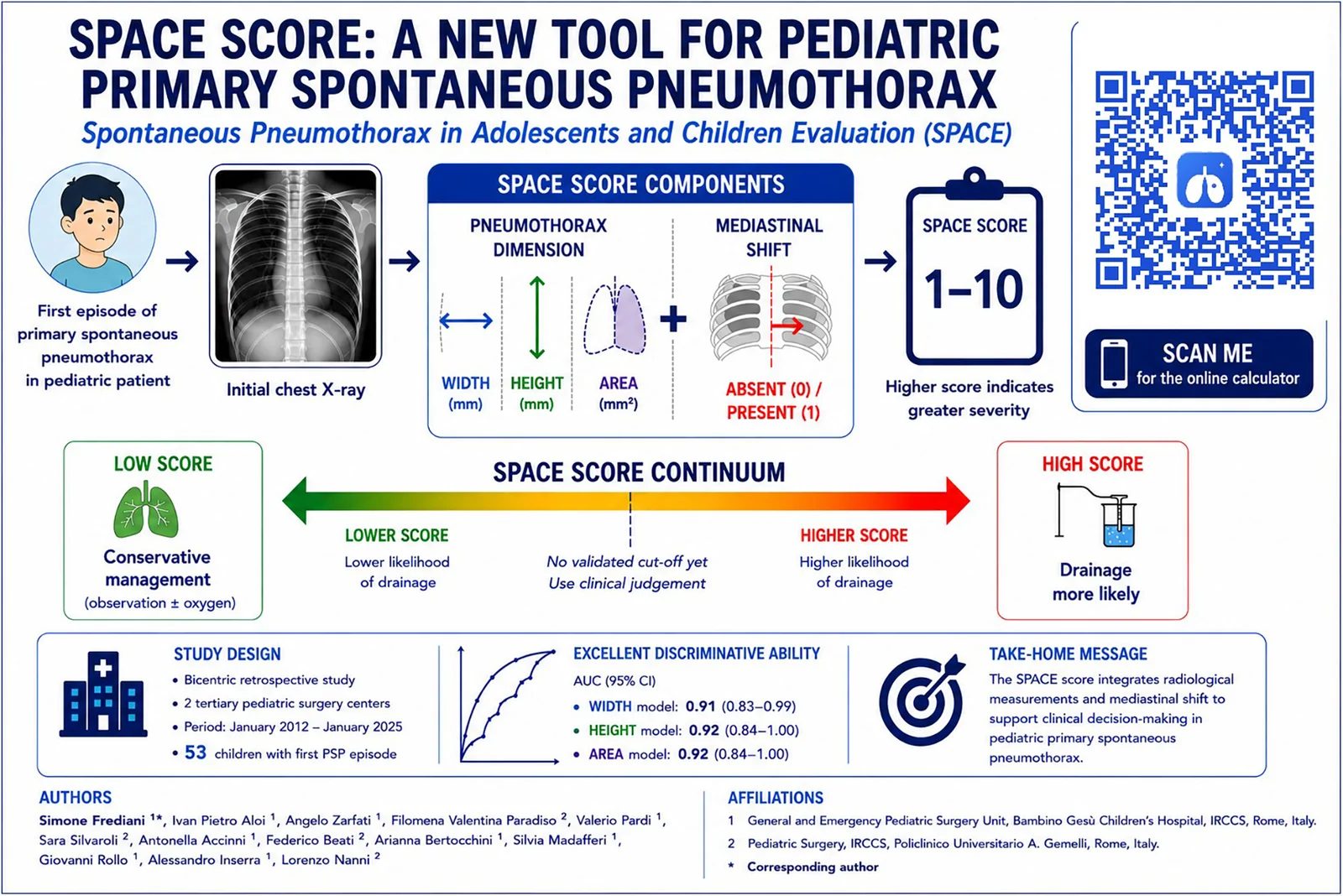

## Introduction

Primary spontaneous pneumothorax (PSP) is a pneumothorax that develops in the absence of trauma or lung parenchymal conditions (1, 2). An increased incidence has been reported in tall and slim adolescents (3). Female patients are less affected than male individuals, with a ratio of 1:4 (2, 3).

The current evidence in the paediatric population is mostly from retrospective observational studies (1–4). These studies have reported several important differences in diagnosis and treatment of PSP in paediatric patients and in follow-up durations (3, 4). Several national and international guidelines exist for PSP diagnosis and treatment in adult patients (5–7). Currently, no age-specific management recommendations or guidelines exist for paediatric PSP (1, 2, 4, 8–11). Therefore, management of paediatric PSP is still based on adult guidelines and evidence. However, this application may be inappropriate because these guidelines have never been validated for paediatric patients. The divergence between adult-based recommendations and paediatric-specific requirements poses continual challenges in clinical decision-making.

Furthermore, no scoring system has been developed and validated for the diagnosis and management of paediatric PSP (2). To the authors’ best knowledge, the applicability and utility of such a tool has never been studied or reported for paediatric PSP. Our hypothesis was that the development of a scoring system may help clinicians and surgeons in managing PSP in paediatric patients. Therefore, we developed a new tool and named it the Spontaneous Pneumothorax in Adolescents and Children Evaluation (SPACE) score.

Due to the lack of paediatric-specific consensus-based recommendations or guidelines, the SPACE score may assist in shaping future consensus agreements. Contemporary adult guidelines, such as those from the British Thoracic Society (5), ERS/ESTS/ESACTS collaboration (6), and French collaboration guidelines (7), advocate for treatment protocols primarily determined by symptom severity and/or pneumothorax dimensions. Nonetheless, these suggestions are based on adult anatomy and pathophysiology, which may not fully represent paediatric PSP dynamics. Children have more pliable chest walls and may respond differently to changes in intrathoracic pressure, affecting clinical presentation and radiological findings (12).

This study aimed to develop, report and propose a new scoring system (the SPACE score) based on clinical and radiological factors influencing decision-making for the management and treatment of paediatric PSP.

## Methods

### Study design and population

We performed a bicentric study, involving two tertiary paediatric surgery centres located in Rome, Italy. A retrospective review of consecutive pediatric patients with PSP between January 2012 and January 2025 was undertaken. Clinical, radiological, and surgical data, follow-up and outcomes were revised. The number of cases managed at our Institutions during the study period determined the sample size. Locally and nationally referred patients composed the study population. There was no randomization, and management was determined by the surgeon's discretion (13–15).

Patients were eligible for inclusion if they presented with a first episode of PSP in one of the two institutions and had a chest radiograph available upon arrival. Exclusion criteria included second episode of PSP, chest radiograph at the time of hospital admission unavailable, traumatic or non-primary or non-spontaneous pneumothorax, lack of data, being lost to follow-up and older than 18 at the time of diagnosis.

### Data analysis and statistical methodology

Statistical analyses were conducted using IBM SPSS software for Windows (Version 28.0; IBM Corp., Armonk, NY, USA) and R software (Version 4.3.2; R Foundation for Statistical Computing, Vienna, Austria).

Data visualisation, including box plots and histograms, was performed using the ggplot2 package in R. Initially, descriptive statistics, including means and standard deviations for continuous variables and frequencies with percentages for categorical variables was used to summarise patient characteristics. Bivariate analyses were conducted using Fisher's exact test for categorical variables and the Mann–Whitney U test for continuous variables, owing to the non-normal distribution of the data, to compare treatment groups (conservative vs. drainage).

Pneumothorax width and height were measured directly on the initial chest radiograph, and pneumothorax area was calculated as the hemithorax area minus the lung area (Figure 10). Spearman's correlation analysis was performed to assess the relationships between pneumothorax dimensions, mediastinal shift, and treatment type. Variables exhibiting high collinearity were examined to determine their effect on multivariable models. To identify predictors of drainage treatment, binary logistic regression analysis was conducted, with three separate models incorporating pneumothorax width, height, or mediastinal shift. The results were reported as odds ratios (OR) with 95% confidence intervals (CIs). Model fit was evaluated using Nagelkerke's R^2^, Cox & Snell R^2^, and the −2 Log-likelihood ratio test.

**Figure 1:**
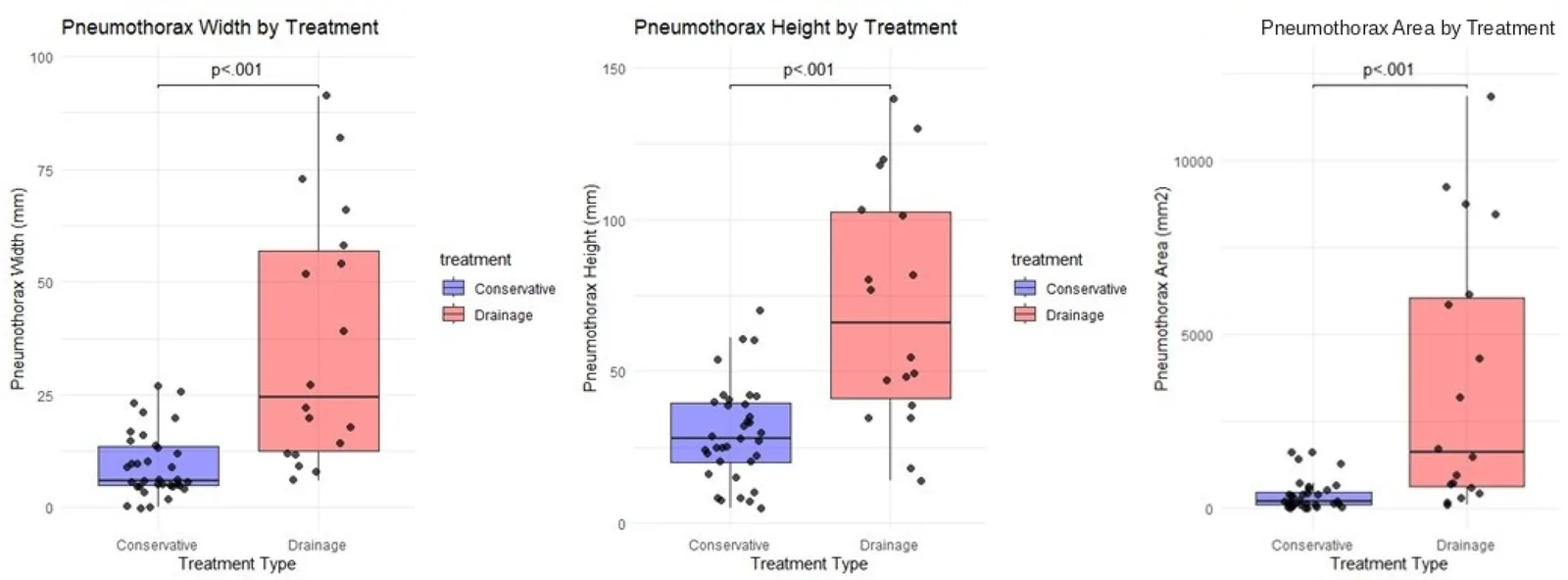
Comparison of pneumothorax dimensions by treatment type. The three boxplots (width, height and area) are displayed side by side, panels A–C, for direct visual comparison. The boxplots illustrate the distribution of pneumothorax width (mm), pneumothorax height (mm), and pneumothorax area (mm^2^) by treatment type (Conservative vs. Drainage). Black dots represent individual data points. The *p*-values (<.001) indicate statistically significant differences between the two treatment groups for each pneumothorax dimension. Conservative treatment is shown in blue, while drainage treatment is shown in red.

To develop a clinical scoring system, probability estimates from the logistic regression models were transformed into a standardised scale of 1–10. The validity of the derived scores was assessed using Mann–Whitney U tests to compare score distributions between the treatment groups. Finally, receiver operating characteristic (ROC) curve analysis was performed to evaluate the predictive accuracy of the computed scores in distinguishing between conservative and drainage treatments. The area under the ROC curves (AUCs) were reported with their 95% CIs, and higher AUC values indicated superior predictive performance.

All statistical tests were two-tailed, with a significance level set at *p* *<* 0.050.

## Results

The study population consisted in 53 paediatric patients diagnosed with PSP. The mean age at the first episode was 14.1 years [standard deviation (*SD*) = 3.3]. The majority of the sample consisted of male patients (*n* *=* 41, 77%) while female patients accounted for 23% (*n* *=* 12). Patients were categorised into two treatment groups based on their initial management: conservative (*n* *=* 35, 66%) and drainage (*n* *=* 18, 34%) treatments. The details of the study population (overall and each treatment group) are presented in Table 1

**Table 1:** Demographic, clinical, and radiological characteristics of patients by treatment type. Values are presented as Mea*n* ± SD for normally distributed continuous variables and Median (IQR) for non-normally distributed variables. Comparisons between treatment groups were conducted using Fisher's exact test for categorical variables and Mann–Whitney U tests for continuous variables. Variables marked with † indicate the presence of missing values. .

Variables	Total Sample (*n* *=* 53)	Treatment Type		*p value*
		Conservative (*n* *=* 35)	Drainage (*n* *=* 18)	
Males, *n (%)*	41 (77.4)	27 (77.1)	14 (77.8)	
^†^Symptomatic, *n (%)*	47 (97.9)	33 (100)	14 (93.3)	.313
^†^Present, PSP, *n (%)*	15 (31.3)	4 (12.1)	11 (73.3)	<.001
Age, *year*		.627		
*M* *±* *SD*	14.1 *±* 3.3	14.1 *±* 3.6	14.2 *±* 2.7	
Median (IQR)	15 (13–16)	15 (14–16)	14.5 (13–16)	
^†^BMI, *kg/m^2^*			.240	
*M* *±* *SD*	18.5 *±* 3.1	18.6 *±* 2.4	18.1 *±* 4.4	
Median (IQR)	17.8 (16.6–19.6)	18.1 (16.9–19.8)	17.3 (15.9–19.6)	
^†^SpO2, *%*			.076	
*M* *±* *SD*	98.4 *±* 2.7	98.8 *±* 1.7	97.5 *±* 3.9	
Median (IQR)	99 (98–100)	100 (98–100)	98 (97–100)	
^†^Heart Rate, *bpm*				.131
*M* *±* *SD*	85.5 *±* 20.0	83.2 *±* 19.3	90.8 *±* 21.3	
Median (IQR)	82 (72–99)	80 (70–90)	94 (72–108)	
^†^Respiratory Rate, *bpm*			.348	
*M* *±* *SD*	19.7 *±* 5.2	20.3 *±* 5.8	18.4 *±* 3.0	
Median (IQR)	19 (17–21)	19.5 (17–21)	18 (16–21)	
Pneumothorax Width, *mm*			<.001	
*M* *±* *SD*	18.7 *±* 21.5	9.4 *±* 7.2	36.8 *±* 28.0	
Median (IQR)	10 (5–21)	6 (5–14)	24.5 (12–58)	
Pneumothorax Height, *mm*			<.001	
*M* *±* *SD*	43.9 *±* 32.9	29.6 *±* 16.0	71.7 *±* 39.5	
Median (IQR)	35 (23–54)	28 (20–40)	66 (39–103)	
Pneumothorax Area, *mm*^2^			<.001	
*M* *±* *SD*	1470.5 *±* 2699.9	374.9 *±* 452.2	3600.8 *±* 3822	
Median (IQR)	400 (125–1281)	174 (75–504)	1589 (588–6136)	
Length of Hospital Stay, *days*				<.001
*M* *±* *SD*	7.9 *±* 7.9	5.2 *±* 5.7	13.2 *±* 8.9	
Median (IQR)	6 (3–10)	4 (2–6)	10 (7–16)	

### Bivariate analyses

Bivariate analyses were conducted to compare demographic, clinical, and pneumothorax-related characteristics between the conservative and drainage groups. The analyses results are presented in Table 1. Sex distribution was similar between the groups (*p* *=* .999, *φ* = .01). We found no significant association between sex and treatment type [*RR* = 1.01, 95% CI (0.64, 1.60)].

Overall, the symptomatic status did not significantly differ between the groups (*p* *=* .313, *φ* = .22), with 97.9% (*n* *=* 47) of patients being symptomatic. However, mediastinal shift was significantly more common in the drainage group than in the conservative group (*p* *<* .001, *φ* = .61). In the drainage group, 73.3% of the patients had mediastinal shift, compared to 12.1% in the conservative group. Furthermore, patients with mediastinal shift had 3.2 times the risk of requiring drainage [*RR* = 3.2, 95% CI (1.41, 7.70)]. The median age at first episode was 15 years [interquartile range (*IQR*): 13–16 years], with no significant differences between the groups [*U*(53) = 289.5, *z* = −0.49, *p* *=* .627]. Similarly, body mass index was not significantly different [*U*(47) = 188.5, *z* = −1.18, *p* *=* .240], with a median of 17.8 kg/m^2^ (*IQR*: 16.6–19.6 kg/m^2^) in our population. Although oxygen saturation (SpO₂, %) at presentation of PSP seemed slightly lower in the drainage group than in the conservative group; the difference was not statistically significant [*U*(51) = 205.5, *z* = −1.78, *p* *=* .076]. Likewise, heart rate (bpm) [*U*(49) = 185.5, *z* = −1.51, *p* *=* .131] and respiratory rate (bpm) [*U* (44) = 173.0, *z* = −0.94, *p* *=* .348] did not differ significantly between the groups.

Regarding the analysis of the PSP size, the patients in the drainage group had a significantly larger size than that of those in the conservative group. Pneumothorax width was significantly more [*U*(53) = 92.0, *z* = −4.20, *p* *<* .001] in the drainage group (*M* = 36.83 ± 28.05 mm, *Mdn* *=* 24.50 mm, IQR: 12–58 mm) than in the conservative group (*M* = 9.46 ± 7.27 mm, *Mdn* *=* 6 mm, IQR: 5–14 mm). Similarly, the pneumothorax height was larger in the drainage group (*M* = 71.72 ± 39.50 mm, *Mdn* *=* 66 mm, IQR: 39–103 mm) than in the conservative group (*M* = 29.63 ± 16.09 mm, *Mdn* *=* 28 mm, IQR: 20–40 mm), showing a significant difference [*U*(53) = 104.0, *z* = −3.96, *p* *<* .001]. The pneumothorax area was significantly greater in the drainage group (*M* = 3600.83 ± 3822 mm^2^, *Mdn* *=* 1589 mm^2^, IQR: 588–6136 mm^2^) than in the conservative group (*M* = 374.97 ± 452.26 mm^2^, *Mdn* *=* 174 mm^2^, IQR: 75–504 mm^2^), with a significant difference observed [*U*(53) = 93.0, *z* = −4.17, *p* *<* .001].

Furthermore, the length of hospital stay was significantly longer in the drainage group (*M* = 13.28 ± 8.96 days, *Mdn* *=* 10 days, IQR: 7–16 days) than in the conservative group (*M* = 5.23 ± 5.77 days, *Mdn* *=* 4 days, IQR: 2–6 days), with a statistically significant difference [*U*(53) = 80.0, *z* = −4.43, *p* *<* .001].

### Correlation between clinical and radiographic characteristics

Spearman's correlation coefficients were calculated separately for the conservative (*n* *=* 35) and drainage (*n* *=* 18) treatment groups to examine the associations between SpO₂ (%), heart rate (bpm), respiratory rate (bpm), pneumothorax dimensions (width, height, and area), and length of hospital stay (days). The findings are presented in Table 2.

**Table 2:** Spearman's correlation matrix for clinical and radiological variables by treatment type. Spearman's rho correlation coefficients are reported for each treatment group (Conservative and Drainage). Correlations assess the relationships between treatment type, 1 = oxygen saturation (SpO₂, %), 2 = heart rate (bpm), 3 = respiratory rate (bpm), 4 = pneumothorax width (mm), 5 = pneumothorax height (mm), 6 = pneumothorax area (mm^2^), and 7 = length of hospital stay (days). * Indicates *p* < .05 and ** indicates *p* < .01.

Variables	1	2	3	4	5	6	7
Conservative *(n* *=* *35)*							
Oxygen Saturation	1						
Heart Rate	−.40*	1					
Respiratory Rate	−.37*	.57**	1				
Pneumothorax Width	.29	−.14	−.16	1			
Pneumothorax Height	.28	−.22	−.10	.82**	1		
Pneumothorax Area	.30	−.18	−.14	.96**	.92**	1	
Length of Hospital Stay	.23	.29	.14	.47**	.24	.42*	1
Drainage *(n* *=* *18)*							
Oxygen Saturation	1						
Heart Rate	.18	1					
Respiratory Rate	.11	−.21	1				
Pneumothorax Width	−.33	.08	.04	1			
Pneumothorax Height	−.33	.28	.03	.94**	1		
Pneumothorax Area	−.34	.19	−.02	.98**	−.97**	1	
Length of Hospital Stay	.05	.17	.16	−.23	−.11	−.17	1

In the conservative group, significantly correlated with length of hospital stay (*ρ* = 0.42, *p* *=* .01), suggesting that a larger pneumothorax is associated with prolonged hospitalisation.

In the drainage group, correlations were generally weaker and did not reach statistical significance. Unlike the conservative group, length of hospital stay did not show a significant relationship with pneumothorax dimensions in the drainage group.

**Figure 2:**
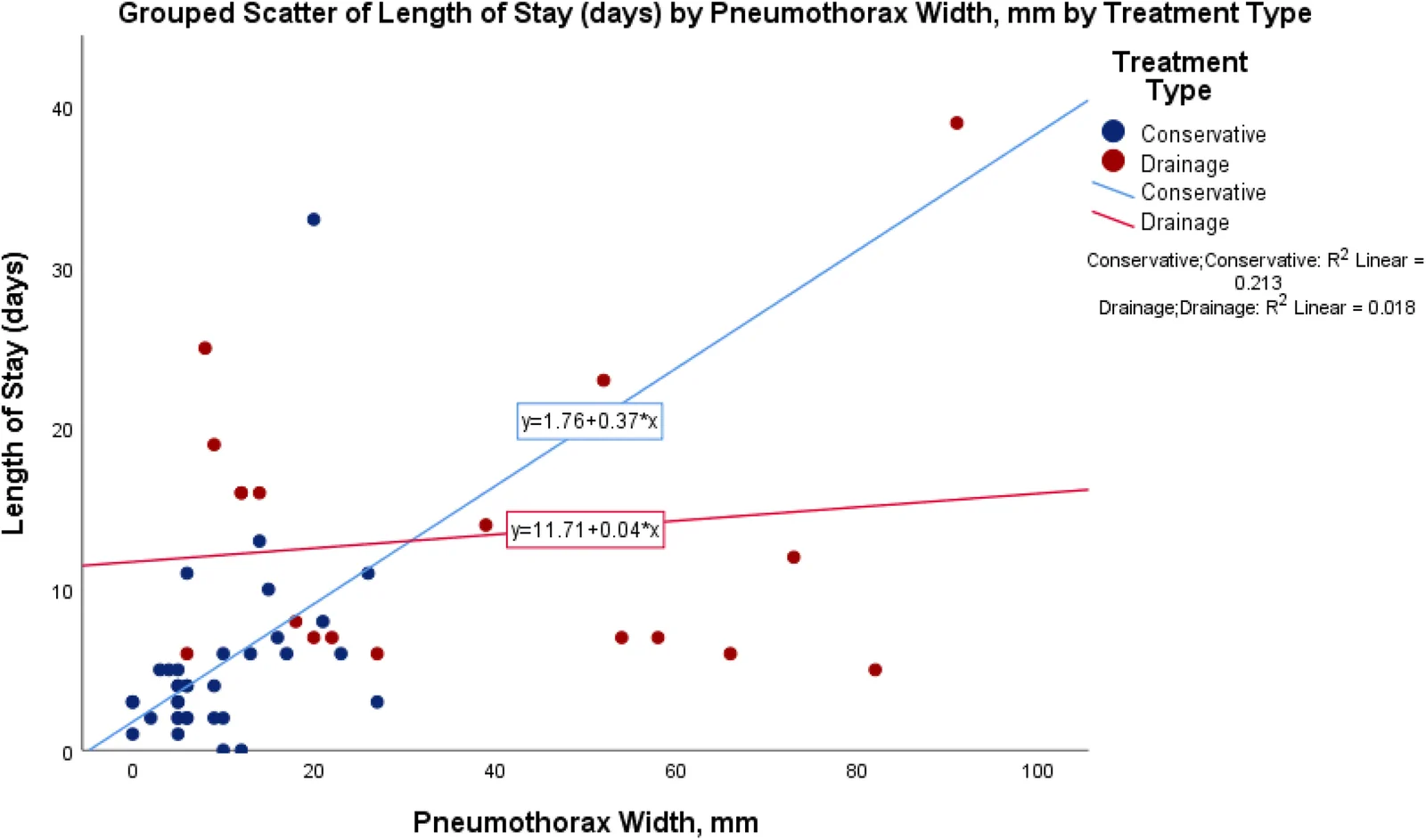
Scatter plot of length of hospital stay (days) by pneumothorax width (mm) across treatment types. This scatter plot illustrates the relationship between pneumothorax width (mm) and length of hospital stay (days) in patients receiving conservative or drainage treatment. The fitted regression lines indicate the association between pneumothorax width and hospital stay duration for each treatment type.

**Figure 3:**
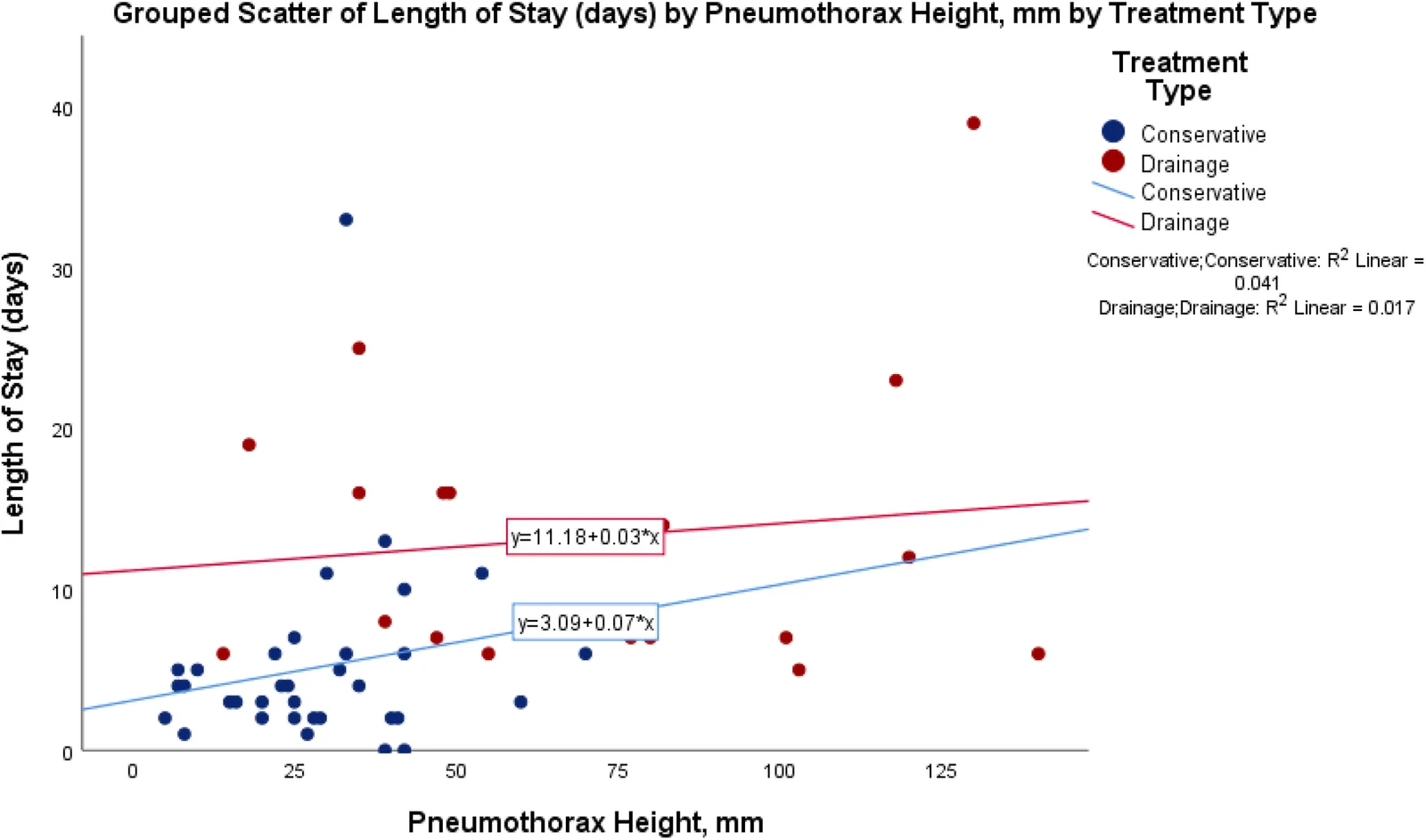
Scatter plot of length of hospital stay (days) by pneumothorax height (mm) across treatment types. This scatter plot shows the association between pneumothorax height (mm) and length of hospital stay (days), stratified by treatment type. The linear regression lines represent the trends observed in the conservative and drainage groups.

**Figure 4:**
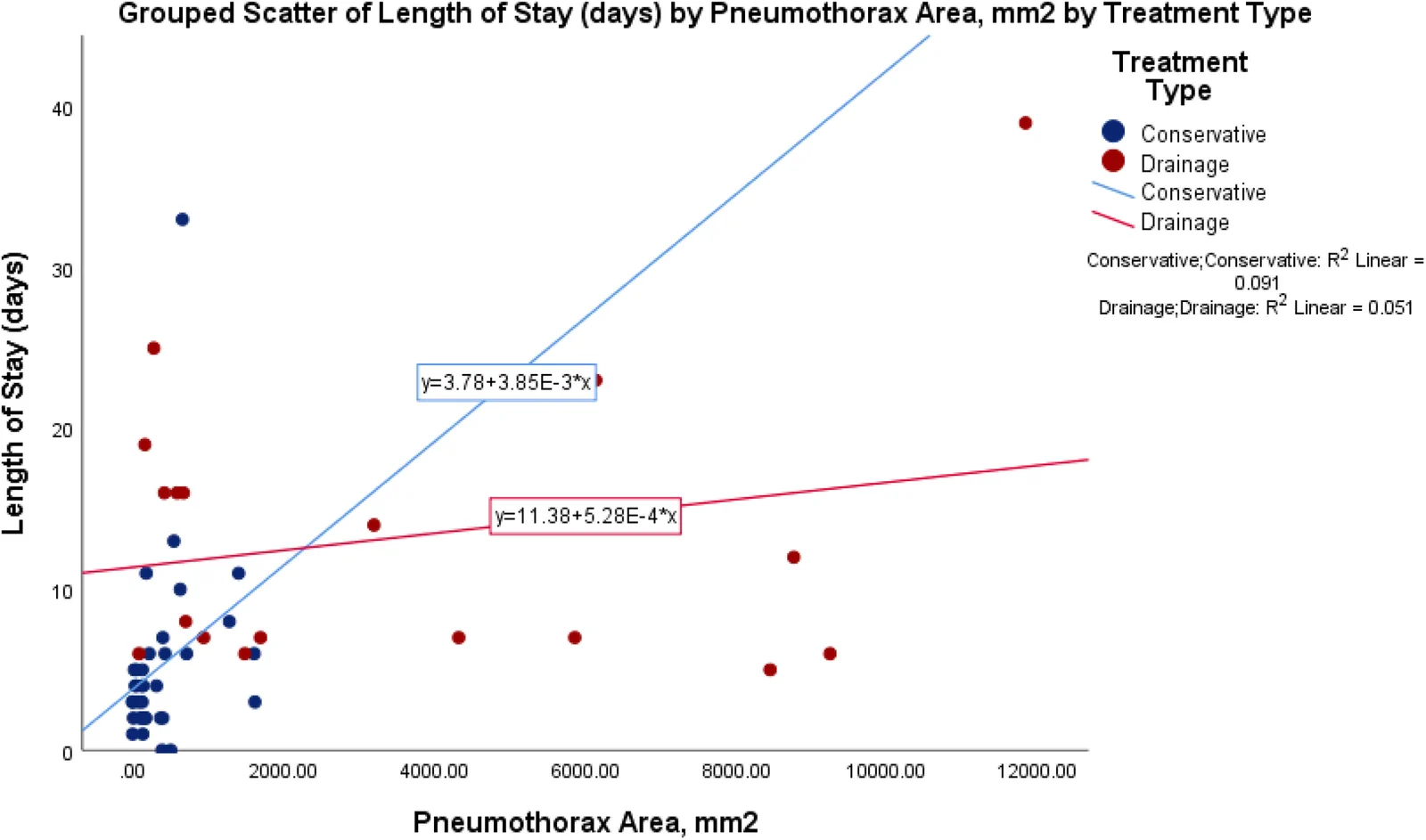
Scatter plot of length of hospital stay (days) by pneumothorax area (mm^2^) across treatment types. This scatter plot examines the relationship between pneumothorax area (mm^2^) and length of hospital stay (days). The regression lines demonstrate how the pneumothorax area influences hospitalisation duration among patients undergoing conservative and drainage treatments.

The scatters plots in Figures 1–4 illustrate the relationships between PSP dimensions (width, height, and area) and length of hospital stay (days) between treatment groups. Figure 2 shows that pneumothorax width was positively correlated with length of hospital stay, with a stronger association in the conservative group, whereas the drainage group exhibited a weaker trend. Similarly, Figure 3 demonstrates that the pneumothorax height had a weaker association with length of hospital stay, with only a slight increase in length of hospital stay as height increased, particularly in the conservative group. Figure 4 indicates that the pneumothorax area had a stronger correlation with length of hospital stay, suggesting that a large pneumothorax was associated with prolonged hospitalisation, especially in the conservative group. Overall, the width and area appeared to be better predictors of hospitalisation duration than pneumothorax height was, and these relationships were more pronounced among patients managed conservatively.

### Binary logistic regression analysis

We performed a binary logistic regression analysis to examine the association between mediastinal shift and pneumothorax dimensions [width [model 1], height [model 2], and area [model 3]] with the likelihood of requiring drainage treatment. The analysis is presented in detail in Table 3.

**Table 3:** Binary logistic regression models predicting the likelihood of drainage treatment based on the presence of mediastinal shift and pneumothorax dimensions.

	95% CI for *Exp (b)*					
Models	*b*	*SE*	Wald	*Exp (b)*	Lower	Upper
Model 1						
*Intercept*	−3.27	.86	14.53	.04***		
Mediastinal shift occurrence	1.83	.96	3.66	6.23*	0.95	40.57
Pneumothorax Width	0.10	.05	4.75	1.10*	1.01	1.21
Model 2						
*Intercept*	−4.01	1.09	13.58	.02***		
Mediastinal shift occurrence	1.86	.94	3.92	6.40*	1.02	40.11
Pneumothorax Height	0.05	.02	5.99	1.06*	1.01	1.10
Model 3						
*Intercept*	−2.67	.69	14.95	.07		
Mediastinal shift occurrence	1.96	.96	4.22	7.14*	1.09	46.55
Pneumothorax Area	.001	.001	2.76	1.01	1.00	1.00

*n* = 48. Dependent variable: Treatment type. b represents regression coefficient. SE indicates standard error of b. Exp (b) indicates the odds ratio. * Indicates *p* < .05 and ** indicates *p* < .001.

Model 1, which included mediastinal shift and pneumothorax width, was statistically significant [*X^2^*(2) = 28.77, *p* *<* .001], with a −2 log-likelihood of 30.85, Cox & Snell *R^2^* of 0.45, and Nagelkerke *R^2^* of 0.63, correctly classifying 85.4% of cases. In this model, the presence of a mediastinal shift increased the likelihood of requiring drainage [*p* *=* .05, *OR* = 6.23, 95% CI (0.95, 40.57)], although the CI was wide, while pneumothorax width was a significant predictor [*p* *=* .029, *OR* = 1.10, 95% CI (1.01, 1.21)], indicating that for every 1 mm increase in width, the odds of requiring drainage increased by 10%.

Model 2, which included mediastinal shift and pneumothorax height, was also statistically significant [*X^2^*(2) = 27.91, *p* *<* .001], with a −2 log-likelihood of 31.71, Cox & Snell *R^2^* of 0.44, and Nagelkerke *R^2^* of 0.62, correctly classifying 83.3% of cases. In this model, presence of mediastinal shift remained a significant predictor of requiring drainage [*p* *=* .048, *OR* = 6.40, 95% CI (1.02, 40.11)], and pneumothorax height was also a significant predictor [*OR* = 1.06, 95% CI (1.01, 1.10)], suggesting that each 1 mm increase in height increased the odds of drainage by 6%.

Model 3, which included mediastinal shift and pneumothorax area, was statistically significant [*X^2^*(2) = 28.63, *p* *<* .001], with a −2 log-likelihood of 30.99, Cox & Snell *R^2^* of 0.45, and Nagelkerke *R^2^* of 0.63, classifying 87.5% of cases correctly. In this model, presence of mediastinal shift still significantly predicted drainage treatment [*p* *=* .040, *OR* = 7.14, 95% CI (1.09, 46.55)], whereas pneumothorax area did not reach statistical significance [*p* *=* .097, *OR* = 1.01, 95% CI (1.00, 1.00)], indicating that the pneumothorax area alone was not an independent predictor.

These findings suggest that a combination of the presence of mediastinal shift and pneumothorax width or height can provide better guidance for determining the necessity for drainage treatment in paediatric PSP cases.

To develop a clinical scoring system for predicting the need for drainage in paediatric PSP, three logistic regression models were utilised. These models estimated the probability of requiring drainage based on mediastinal shift and different pneumothorax dimensions (width, height, and area). The probability (p) obtained from each model was transformed into scaled score ranging from 1 to 10, where higher scores indicated a greater likelihood of requiring drainage. Logistic regression equations were used to estimate *p*, with Model 1 incorporating pneumothorax width, Model 2 incorporating pneumothorax height, and Model 3 incorporating pneumothorax area (Table 3).

The probability was then converted into a clinical score using the formula

- Regression Equation for Model 1

Log (*p*/1-*p*)=−3.27 + (1.83*mediastinal shift) + (0.10*pneumothorax width)

$$Score\;1=10*\left(\frac{\exp\;(-3.27+1.83\;*\;\mathrm{mediastinal}\;\mathrm{shift}+0.10\;*\;\mathrm{Pneumothorax}\;\mathrm{Width})}{\;(1+\exp\;(-3.27+1.83\;*\;\mathrm{mediastinal}\;\mathrm{shift}\;+0.10\;*\;\mathrm{Pneumothorax}\;\mathrm{Width}))}\right)$$

- Regression Equation for Model 2

Log (*p*/1-*p*)=−4.01+ (1.86*mediastinal shift) + (0.05*pneumothorax height)

$$Score\;2=10*\left(\frac{\exp\;(-4.01+1.86\;*\;\mathrm{mediastinal}\;\mathrm{shift}+0.05\;*\;\mathrm{Pneumothorax}\;\mathrm{Height})}{\;(1\;+\exp\;(-4.01+1.86\;*\;\mathrm{mediastinal}\;\mathrm{shift}+0.05\;*\;\mathrm{Pneumothorax}\;\mathrm{Height}))}\right)$$

- Regression Equation for Model 3

Log (*p*/1-*p*)=−2.67+ (1.96* mediastinal shift) + (0.001*pneumothorax area)

$$Score\;3=10*\left(\frac{\exp\;(-2.67+1.96\;*\;\mathrm{mediastinal}\;\mathrm{shift}+0.001\;*\;\mathrm{Pneumothorax}\;\;\mathrm{Area})}{\;(1+\exp\;(-2.67+1.96*\;\mathrm{mediastinal}\;\mathrm{shift}+0.001\;*\;\mathrm{Pneumothorax}\;\;\mathrm{rea}))}\right)$$

This transformation ensured that the scores are clinically interpretable and provide a standardised framework for risk stratification. Higher scores correspond to an increased probability of requiring drainage, allowing for a more individualised approach to clinical decision-making.

### Validity of a clinical scoring system

To clarify, the SPACE score is not a single combined score: three parallel candidate scores were derived (Score 1, Score 2 and Score 3), each combining mediastinal shift with one radiological dimension (pneumothorax width, height, or area, respectively), so that clinicians can apply whichever measurement is most readily available on the initial chest radiograph. All three showed comparable, excellent discriminative performance (Table 5). A single, formally validated cut-off for recommending drainage has not yet been defined; the score distributions differed clearly between the conservative and drainage groups (see below), but identifying an optimal cut-off (for example, via the Youden index) and determining whether one model, or a further combined model, should be adopted for routine use will require prospective validation.

**Figure 5:**
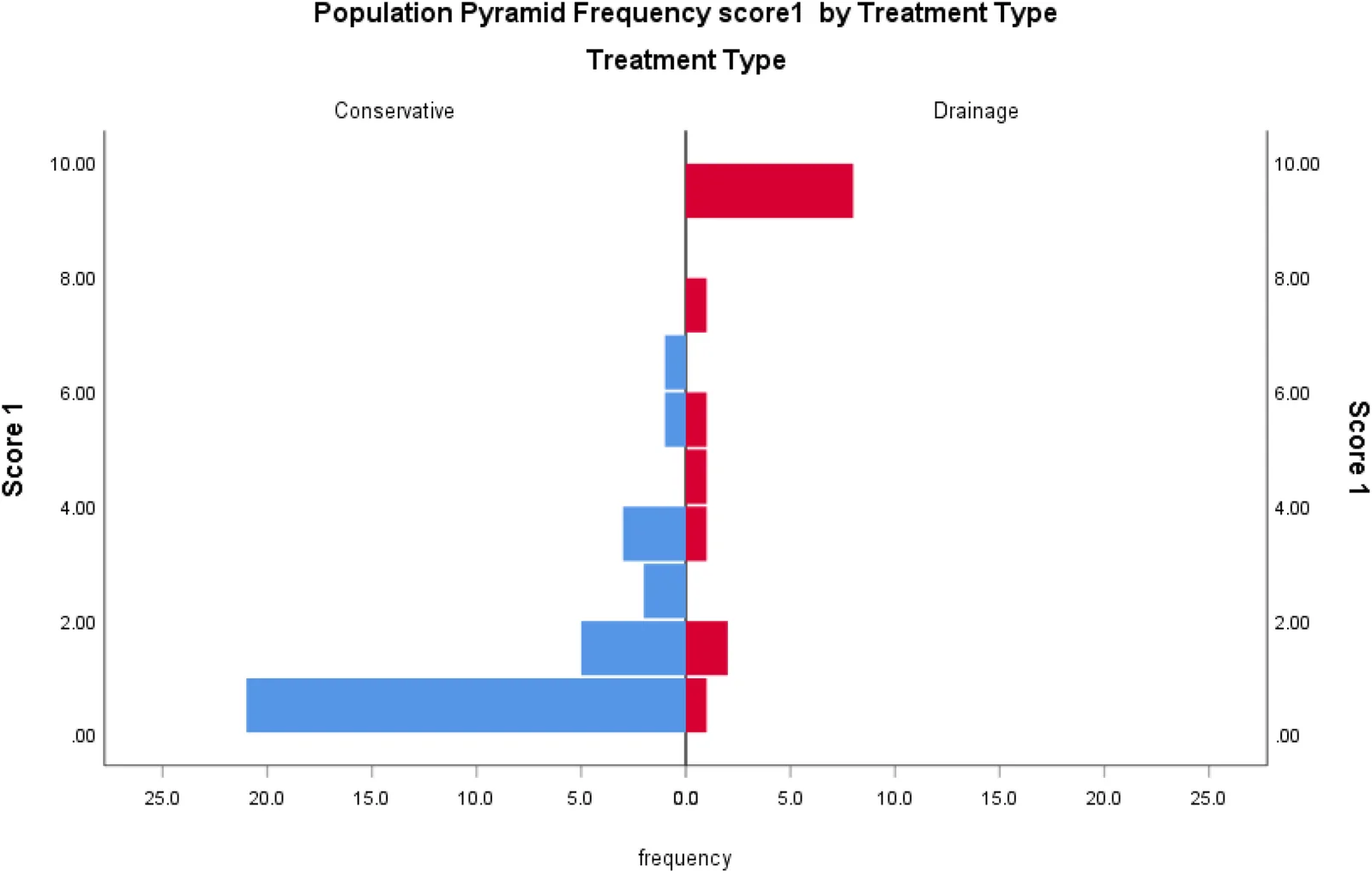
Population pyramid of score 1 distribution by treatment type. Distribution of Score 1, calculated based on pneumothorax width and mediastinal shift, is shown across treatment types. Lower scores are predominantly observed in the conservative treatment group, whereas higher scores are more frequent in the drainage group.

**Figure 6:**
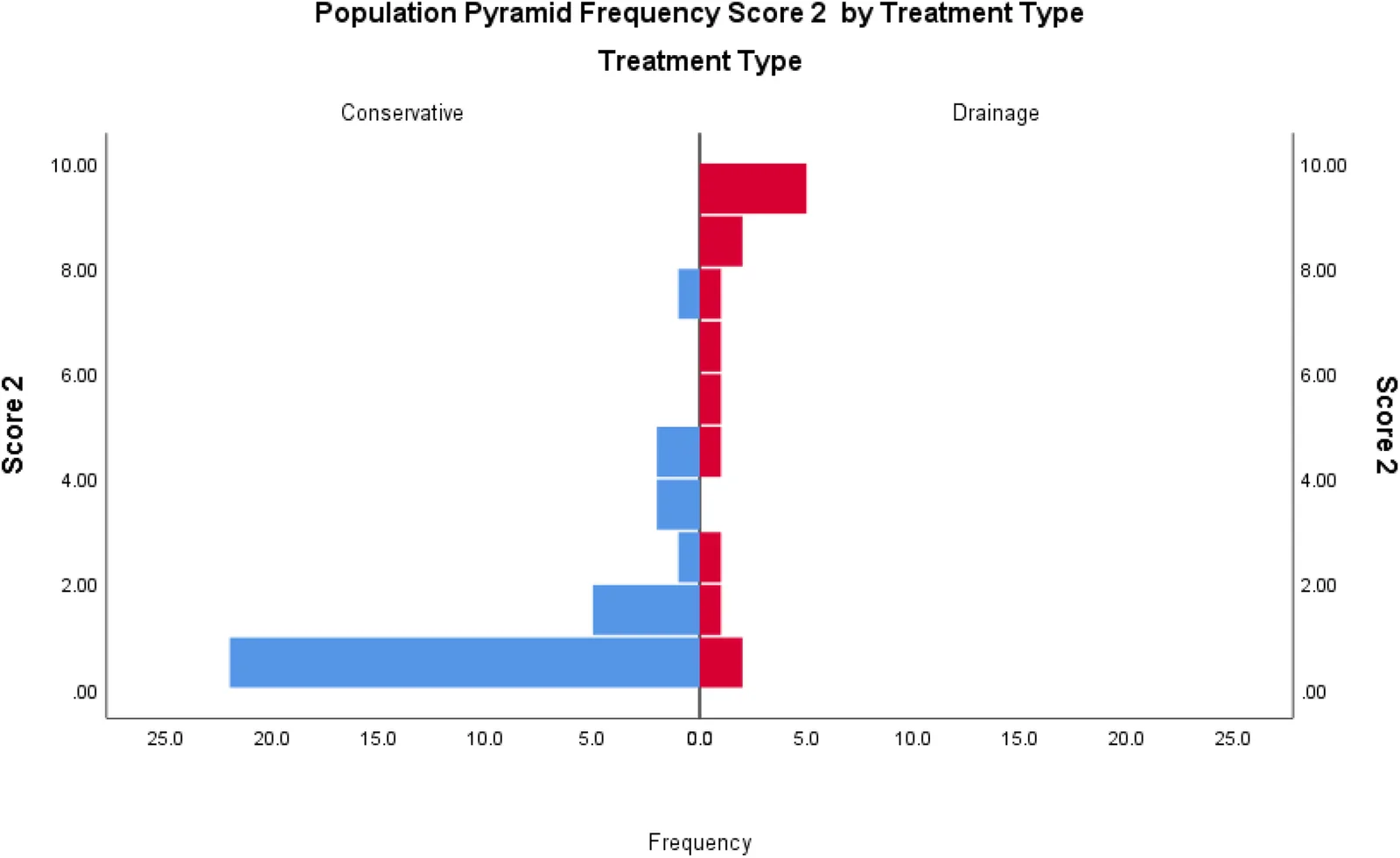
Population pyramid of score 2 distribution by treatment type. Distribution of Score 2, derived from pneumothorax height and PSP, is displayed across treatment types. Lower scores are more common in the conservative treatment group, while higher scores indicate a greater likelihood of requiring drainage treatment.

**Figure 7:**
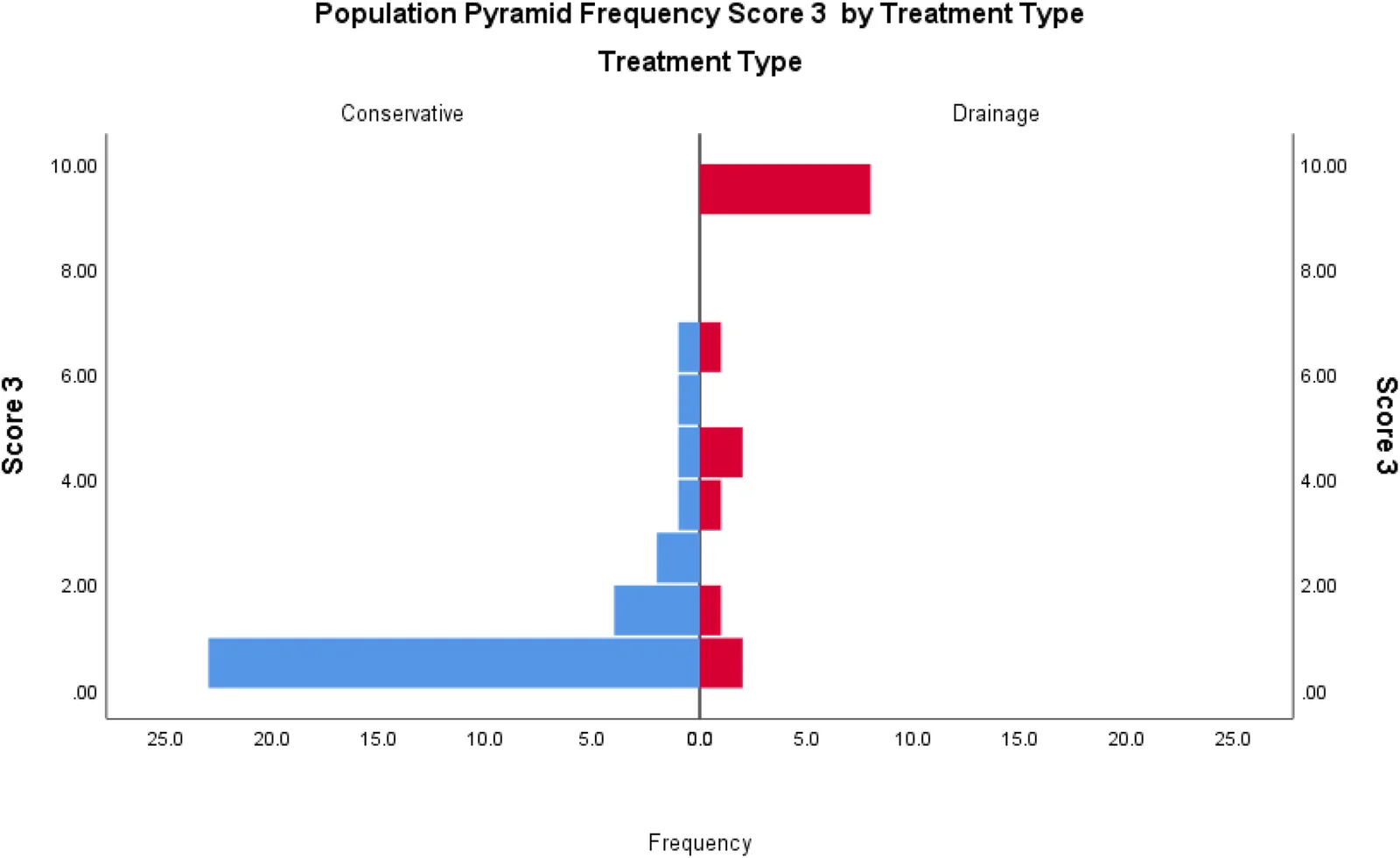
Population pyramid of score 3 distribution by treatment type. Distribution of Score 3, calculated using pneumothorax area and PSP, is illustrated across treatment types. Higher scores are strongly associated with the need for drainage treatment, while lower scores are more frequent in patients managed conservatively.

The distribution of the three calculated scores (Scores 1, 2, and 3) across treatment groups is illustrated in Figures 5–7. These scores were derived from logistic regression models incorporating pneumothorax dimensions (width, height, and area) and the presence of mediastinal shift to assess their predictive capacity for treatment selection.

Figure 5 presents the distribution of Score 1, calculated using pneumothorax width and PSP. Lower scores were predominantly observed in the conservative group, whereas higher scores were more frequent in the drainage group. Figure 6 illustrates the distribution of Score 2, derived from pneumothorax height and presence of mediastinal shift, following a similar pattern, with low scores more commonly observed for conservative treatment and high scores indicating a greater likelihood of drainage treatment. Figure 7 displays Score 3, which incorporates the pneumothorax area and PSP, showing a strong association between higher scores and the need for drainage.

These findings indicate that higher scores, which reflect greater PSP dimensions and the presence of mediastinal shift, are strongly associated with the need for drainage, whereas lower scores are more frequently observed in patients managed conservatively. This scoring system, based on logistic regression equations, provides a quantitative assessment of pneumothorax severity and may assist in clinical decision-making regarding treatment strategies.

Furthermore, the descriptive statistics confirm these trends. Patients in the conservative group had significantly lower mean scores (Score 1: *M* = 1.43, *SD* = 1.52; Score 2: *M* = 1.32, *SD* = 1.61; and Score 3: *M* = 1.39, *SD* = 1.41) than those had by the drainage group (Score 1: *M* = 6.88, *SD* = 3.64; Score 2: *M* = 6.41, *SD* = 3.50; Score 3: *M* = 6.79, *SD* = 3.70).

**Figure 8:**
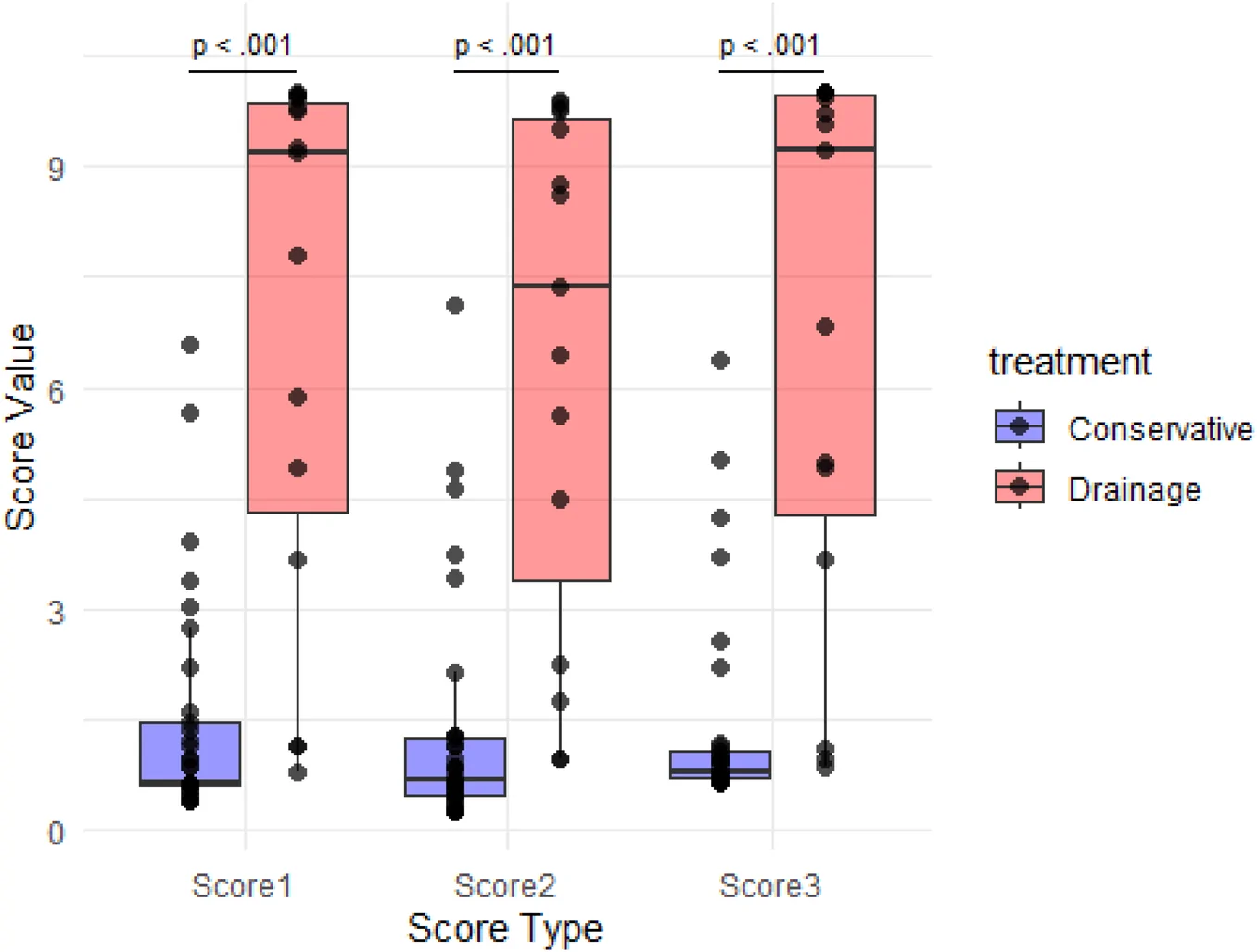
Distribution of clinical scores by treatment type for first PSP episode. The box plot illustrates the distributions of Scores 1, 2, and 3 across conservative (blue) and drainage (red) treatment groups. Each score represents a predictive measure derived from logistic regression models incorporating pneumothorax dimensions (width, height, and area) and mediastinal shift. The median score is significantly higher in the drainage group than in the conservative group (*p* < .001), indicating that higher scores are associated with an increased likelihood of requiring drainage treatment.

The Mann–Whitney U test was conducted to compare the median scores between the conservative and drainage treatment groups (Table 4). The results indicated that patients requiring drainage had significantly higher scores across all the three models compared to those had by patients managed conservatively. Specifically, the median Score 1 was 9.21 (*IQR*=3.68, 9.94) in the drainage group, significantly higher than 0.65 (*IQR*=0.59, 1.46) in the conservative group [*U*(48) = 45.0, *z* = −4.52, *p* *<* .001]. Similarly, the median Score 2 in the drainage group was 7.39 (*IQR* = 2.23, 9.77), compared to 0.68 (*IQR*=0.47, 1.23) in the conservative group [*U*(48) = 39.0, *z* = −4.64, *p* *<* .001]. The median Score 3 followed the same trend, with 9.23 (*IQR* = 3.66, 10.00) in the drainage group and 0.76 (*IQR* = 0.69, 1.07) in the conservative group [*U*(48) = 42.0, *z* = −4.57, *p* *<* .001]. (Figure 8) These findings suggest that higher scores were strongly associated with the need for drainage treatment, reinforcing the predictive utility of the scoring models based on pneumothorax width, height, area, and mediastinal shift.

**Table 4:** Mann–Whitney U test comparing scores between conservative and drainage treatment groups.

Score	Treatment Type				*p*
	Conservative (*n* *=* 33)		Drainage (*n* *=* 15)		
	*M* *±* *SD*	*Mdn* (IQR)	*M* *±* *SD*	*Mdn* (IQR)	
Score 1	1.43 ± 1.52	0.65 (.59, 1.46)	6.88 ± 3.64	9.21 (3.68, 9.94)	<.001
Score 2	1.32 ± 1.61	0.68 (.47, 1.23)	6.41 ± 3.50	7.39 (2.23, 9.77)	<.001
Score 3	1.39 ± 1.41	0.76 (.69, 1.07)	6.79 ± 3.70	9.23 (3.66, 10.00)	<.001

Values are presented as Mean ± SD for normally distributed continuous variables and Median (IQR) for non-normally distributed variables. Mann–Whitney U test was used to compare the distributions of scores between the treatment groups. Low scores were predominantly observed in the conservative treatment group, while high scores were significantly associated with drainage treatment (*p* < .001 for all models). Scores 1, 2, and 3 were derived from logistic regression models incorporating pneumothorax width, height, area, and mediastinal shift.

### Receiver operating characteristic curve analysis

**Figure 9:**
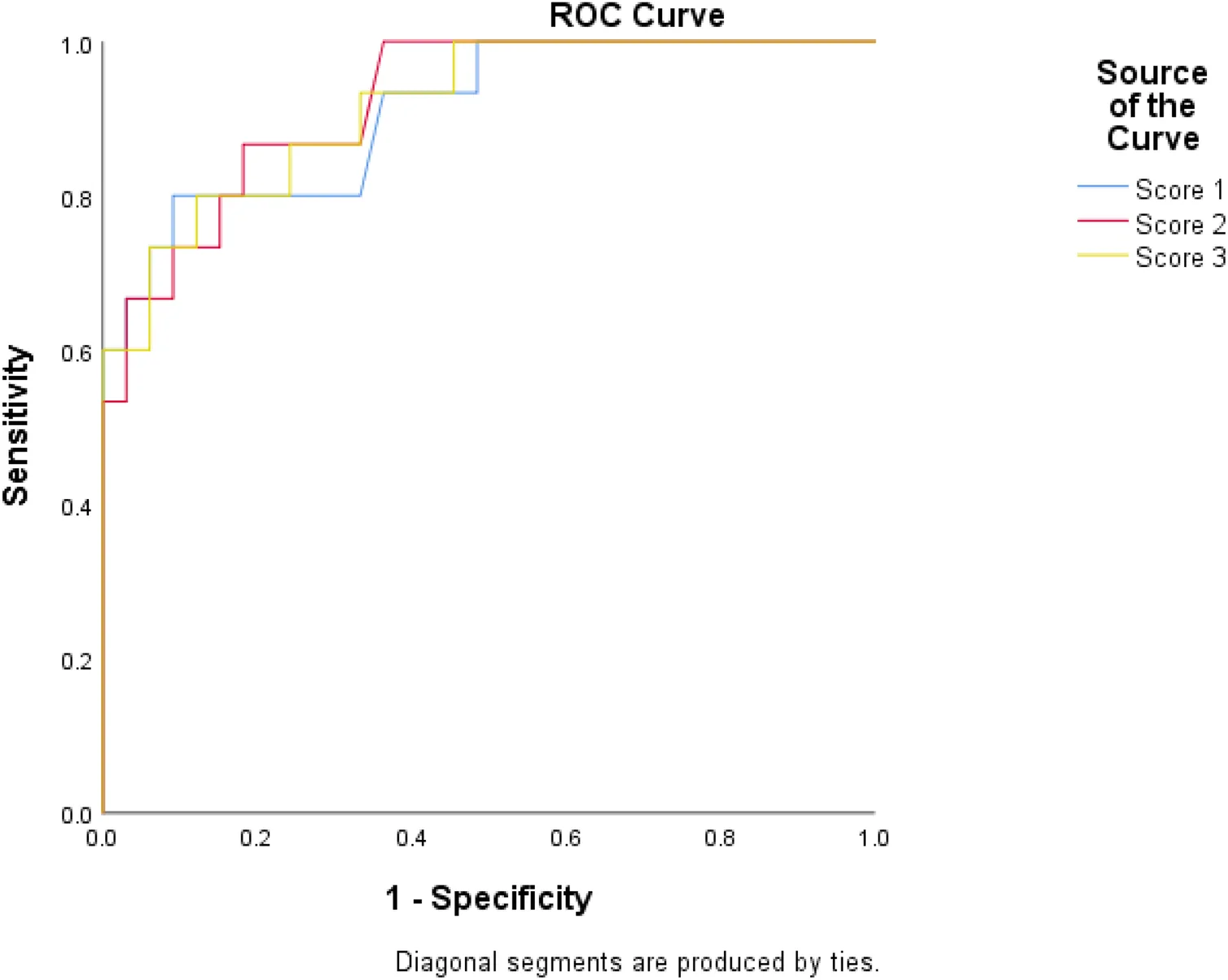
Receiver operating characteristic curve for clinical scores in predicting drainage treatment. The receiver operating characteristic curve displays the predictive performance of the three computed clinical scores (Scores 1, 2, and 3) in determining the need for drainage treatment. The *x*-axis represents 1—Specificity (false positive rate), and the *y*-axis represents Sensitivity (true positive rate). Curves closer to the upper-left corner indicate stronger predictive accuracy. Scores 2 (red line) and 3 (yellow line) demonstrate the highest sensitivity and specificity, while Score 1 (blue line) exhibits excellent discriminatory power. These findings suggest that all the three scoring models provide reliable prediction for drainage treatment decisions in paediatric spontaneous pneumothorax.

ROC curve analysis was conducted to evaluate the predictive accuracy of the developed clinical scores in distinguishing between conservative and drainage treatments for PSP. The analysis results are presented in Table 5 and Figure 9.

**Table 5:** Receiver operating characteristic analysis of clinical scores for predicting drainage treatment in paediatric spontaneous pneumothorax. The Area Under the Curve (AUC) represents the discriminatory ability of each predictor in distinguishing between conservative and drainage treatments. SE denotes the standard error of the AUC estimate. *p*-values indicate the statistical significance of the AUC, with 95% confidence intervals provided for each predictor. AUC values closer to 1.0 indicate stronger predictive accuracy, with values ≥0.80 suggesting good to excellent discrimination.

Test Result Variable(s)	Area	SE^a^	p	95% CI	
				Lower	Upper
Score 1	.91	.05	<.001	.819	.999
Score 2	.92	.04	<.001	.845	.998
Score 3	.92	.04	<.001	.832	.998

Score 1, derived from mediastinal shift and pneumothorax width, demonstrated an AUC of 0.91 [95% CI (0.819, 0.999), *p* < .001], indicating excellent discriminatory power. Similarly, Score 2, incorporating mediastinal shift and pneumothorax height, achieved an AUC of 0.92 [95% CI (0.845, 0.998), *p* < .001], and Score 3, based on mediastinal shift and pneumothorax area, also exhibited strong predictive performance with an AUC of 0.92 [95% CI (0.832, 0.998), *p* < .001].

The ROC curve illustrates the diagnostic performance of the three computed clinical scores (Scores 1, 2, and 3) in predicting the need for drainage treatment.

The *x*-axis (1 - Specificity) represents the false positive rate, while the *y*-axis (Sensitivity) represents the true positive rate, with curves closer to the upper-left corner indicating superior predictive accuracy. Scores 2 (red line) and 3 (yellow line) exhibited the highest sensitivity and specificity respectively, reflecting their strong predictive capability for distinguishing between conservative and drainage treatments. Score 1 (blue line) also demonstrated excellent discrimination, with its curve closely following those of Scores 2 and 3. The similarity in AUC values across the three models suggests that all scoring approaches provide strong predictive accuracy, reinforcing the validity of the proposed clinical scoring system for guiding drainage decisions in PSP.

## Discussion

This study sought to address the divergence between adult recommendations and paediatric-specific requirements by introducing a newly developed and unique quantitative tool —the SPACE score, which was intended to enhance paediatric-specific therapy for PSP. The SPACE score integrates essential radiographic characteristics with the occurrence of mediastinal shift, offering a solid, evidence-driven methodology for management and treatment stratification.

Numerous outcomes from our investigation highlight the clinical significance and prognostic accuracy of the SPACE Score. Initially, we established that the dimensions of pneumothorax, specifically width and height, are significantly correlated with the requirement for drainage intervention. These findings align with prior adult-focused literature, indicating that increased PSP size is associated with clinical worsening and a greater likelihood of interventional management (4). This confirms their pivotal significance in the proposed scoring system.

**Figure 10:**
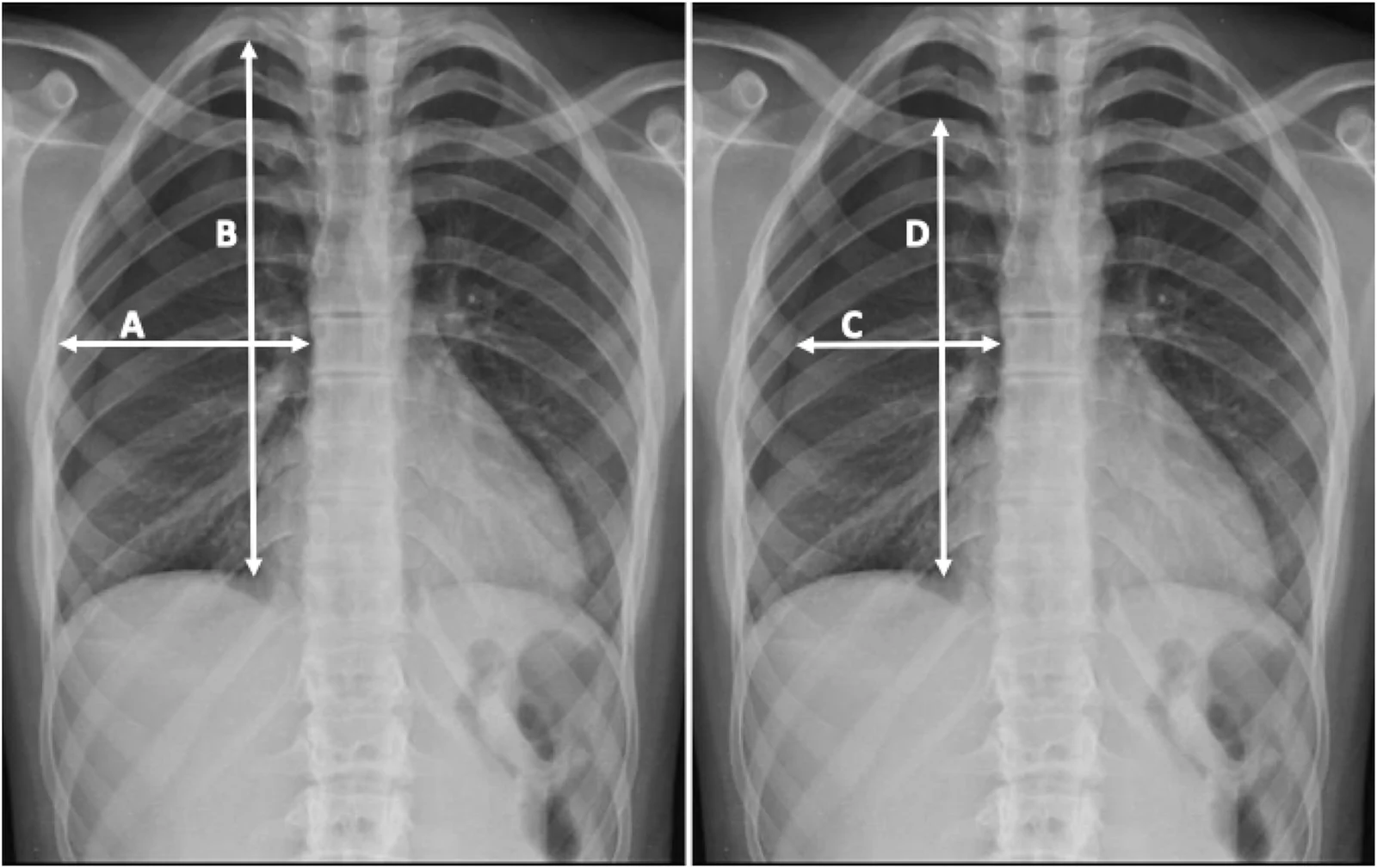
Pneumothorax area calculation. The calculation of the area of the pneumothorax is given by the area of the hemithorax (a × b)—area of the lung (c × d).

Notably, whereas the pneumothorax area (Figure 10) correlated with treatment selection in the univariate analysis, it failed to maintain significance as an independent predictor in the multivariable regression analyses, indicating that width and height may more accurately reflect clinically pertinent severity. This finding corroborates the findings of Miscia et al. (2020), who identified difficulties in quantifying pneumothorax size in paediatric patients owing to anatomical heterogeneity and variations in lung compliance (2).

Our scoring models exhibited superior predictive performance, with all the three variations (based on width, height, and area for the three models, respectively) displaying exceptional discrimination, evidenced by AUC values above 0.90. These findings are especially significant considering the limited cohort size and retrospective methodology. In contrast, adult research employing scoring systems for spontaneous pneumothorax have indicated AUC values between 0.70 and 0.85 (6, 7), implying that the SPACE score may offer enhanced stratification in the paediatric PSP.

A significant and innovative aspect of our research is the incorporation of mediastinal shift as a binary clinical variable. Although typically considered as a radiographic indicator of tension pneumothorax or significant PSP, its function as a predictor of invasive intervention has been minimally assessed in paediatric populations. Our findings demonstrate that mediastinal shift significantly increases the likelihood of drainage treatment by approximately 6–7 times, irrespective of the PSP size. This underscores its potential as a crucial factor in treatment pathways and further advocates for its incorporation into the SPACE score.

The ramifications of our findings surpass risk stratification. The score provides clinicians with a standardised and reproducible approach to facilitate decision-making, decrease interobserver variability, and potentially reduce the need for unnecessary intervention or drainage procedures. In our sample, patients treated conservatively exhibited markedly decreased SPACE scores in all models, corresponding with diminished PSP dimensions and the absence of mediastinal displacement. In contrast, elevated scores reliably indicated the necessity for drainage, a conclusion substantiated by Mann–Whitney U tests and ROC analysis.

The SPACE score may function as a valuable supplement in collaborative decision-making, especially in unclear or borderline situations. It offers a quantitative framework for doctors to utilise while deliberating management options with families, particularly when the choice between observation and action is complex. This is especially pertinent considering the increasing focus on family-centred treatment in paediatric medicine.

Notwithstanding its merits, our study possesses certain limitations. The limited sample size, albeit being sourced from two high-volume tertiary centres, may constrain the generalisability of the results. In addition, as a retrospective, bicentric study, management decisions were made at the discretion of the individual treating surgeon following different institutional protocols; consequently, treatment criteria may have varied between the two participating centres, representing a potential source of heterogeneity that should be considered when interpreting the score's derivation and generalisability. A further important limitation is confounding by indication: patients who underwent drainage were likely already more unwell and had larger pneumothoraces at presentation, and this clinical impression is probably what influenced the treating team's decision at the time, rather than the decision being independent of illness severity. This inherent circularity, common to retrospective studies of treatment selection, means the SPACE score should be interpreted as reflecting the factors historically associated with drainage rather than a causally validated decision rule. Consequently, the SPACE score requires prospective evaluation, ideally across multiple centres with standardised management protocols, before it can be recommended for independent clinical use. Future research should evaluate the score's applicability across diverse populations and healthcare settings, particularly in low-resource contexts where access to advanced imaging and paediatric surgical skills may be constrained.

Moreover, incorporating supplementary clinical parameters—such as symptom severity ratings, underlying comorbidities, and laboratory results—may refine the score and augment its predictive capability. Future multicentric research may investigate whether incorporating the SPACE score into clinical algorithms reduces practice variability, accelerates the time to definitive therapy, and enhances patient outcomes.

## Conclusions

The SPACE score serves as a potential instrument for improving the management of paediatric PSP. By integrating accessible radiological characteristics, it provides an objective, standardised methodology to inform treatment decisions. Our findings endorse its application in categorising patients based on drainage requirements and highlight the necessity for paediatric-specific protocols in PSP management. Subsequent research must prioritise external evaluation and incorporation of the score into therapeutic pathways to enhance its applicability across various clinical settings.

## Data Availability

The raw data supporting the conclusions of this article will be made available by the authors, without undue reservation.
